# Efficacy of Two Vaccination Strategies against Infectious Bronchitis in Laying Hens

**DOI:** 10.3390/vaccines11020338

**Published:** 2023-02-02

**Authors:** Ahmed Ali, Mohamed S. H. Hassan, Shahnas M. Najimudeen, Muhammad Farooq, Salama Shany, Mounir Mohamed El-Safty, Adel A. Shalaby, Mohamed Faizal Abdul-Careem

**Affiliations:** 1 Faculty of Veterinary Medicine, University of Calgary, 3330 Hospital Drive NW, Calgary, AB T2N 4N1, Canada; 2 Department of Pathology, Faculty of Veterinary Medicine, Beni-Suef University, Beni Suef 62511, Egypt; 3 Department of Poultry Diseases, Faculty of Veterinary Medicine, Assiut University, Assiut 71515, Egypt; 4 Department of Poultry Diseases, Faculty of Veterinary Medicine, Beni-Suef University, Beni Suef 62511, Egypt; 5 Central Laboratory for Evaluation of Veterinary Biologics (CLEVB), Abassia, Cairo 11517, Egypt

**Keywords:** infectious bronchitis virus (IBV), vaccination, hens, Canada, Massachusetts (Mass) serotype, Connecticut (Conn) serotype

## Abstract

Vaccination remains the leading control method against infectious bronchitis (IB) in poultry despite the frequently observed IB outbreaks in vaccinated flocks. Here, two vaccination regimes were evaluated against challenge with the Massachusetts (Mass) infectious bronchitis virus (IBV) strain that was linked to egg production defects in Western Canada. One vaccination strategy included live attenuated IB vaccines only, and the other used both inactivated and live attenuated IB vaccines. The two immunization programs involved priming with a monovalent live attenuated IB vaccine (Mass serotype) at day-old, followed by intervals of bivalent live attenuated IB vaccines containing the Mass and Connecticut (Conn) serotypes given to the pullets at 2-, 5-, 9-, and 14-week-old. Inactivated IB vaccine (Mass serotype) was administrated to only one group of the vaccinated birds at 14-week-old. At the peak of lay, the hens were challenged with the Mass IBV isolate (15AB-01) via the oculo-nasal route. The efficacy of the vaccines was assessed following the challenge by observing clinical signs, egg production, egg quality parameters, seroconversion, and systemic T-cell subsets (CD4+ and CD8+ cells). Moreover, the viral genome loads in the oropharyngeal (OP) and cloacal (CL) swabs were quantified at predetermined time points. At 14 days post-infection (dpi), all the hens were euthanized, and different tissues were collected for genome load quantification and histopathological examination. Post-challenge, both vaccination regimes showed protection against clinical signs and exhibited significantly higher albumen parameters, higher anti-IBV serum antibodies, and significantly lower levels of IBV genome loads in OP swabs (at 3 and 7 dpi) and trachea and cecal tonsils compared to the mock-vaccinated challenged group. However, only the birds that received live attenuated plus inactivated IB vaccines had significantly lower IBV genome loads in CL swabs at 7 dpi, as well as decreased histopathological lesion scores and IBV genome loads in magnum compared to the mock-vaccinated challenged group, suggesting a slightly better performance for using live attenuated and inactivated IB vaccines in combination. Overall, the present findings show no significant difference in protection between the two vaccination regimes against the Mass IBV challenge in laying hens.

## 1. Introduction

Infectious bronchitis (IB) is an economically devasting disease in chickens, and its etiological agent is infectious bronchitis virus (IBV) [1]. Initially, IBV targets the tracheal mucosa [1,2]; however, some IBV variants and/or serotypes have been identified in non-respiratory tissues such as kidney [3,4], gastrointestinal tissues [5,6], bursa of Fabricius [6,7], and oviduct [4,8]. It has been shown that IBV can replicate in the lining epithelium of the oviduct leading to diminished egg production and eggshell defects [9]. Globally, IBV is continuously accompanied by high rates of genetic diversity, particularly in its spike1 (S1) gene [10]. The variable degrees of mutation and recombination events together with the host selective pressure can result in IBV variants that may hinder the control of IBV in poultry [11].

IBV is one of the most widespread agents around the world, so it can spread rapidly through chicken flocks, particularly in non-vaccinated birds, which shed the virus in their excretions for long periods [12]. Therefore, the control of IBV through strict biosecurity measures and rearing of birds with uniform ages is often challenging. However, boosting the birds’ immune system through vaccination against IBV infection is critical in its control [13]. Despite the presence of a wide array of novel vaccines against IB, only live attenuated and inactivated vaccines are used commercially [14]. The administration routes of live attenuated vaccines are drinking water, coarse spray, and eye drops [15,16]. Inactivated IB vaccines are usually oil-adjuvanted and administrated via the intramuscular route in layer pullets and breeders. Live attenuated vaccines are usually employed to obtain local immune protection [17,18] and are administrated to prime day-old chicks, followed by multiple boosters to achieve a sustainable mucosal immune response. It was known that the immunization of pullets with intervals of live attenuated IB vaccines followed by boosting with inactivated vaccines 4–6 weeks prior to lay can result in long-lasting immune protection in laying hens [19]. Several vaccination and challenge studies have been conducted using live attenuated vaccines containing different serotypes against distinct IBV challenge strains [20]. Recent studies have revealed that using two heterologous live attenuated IBV vaccines is a much better option to broaden protection against IBV variants than one. This is called the “protectotype” approach. Currently, the protectotype approach is widely used to control IBV outbreaks in chicken flocks globally [21,22].

Although many experimental IBV vaccination/challenge trials have been performed to evaluate the protective efficacy in young chicks, few of these experiments have been conducted in laying birds [23]. The main explanation for this is the cost of rearing of such birds for long periods in well-equipped, isolated, and controlled environments. Most previous studies in the 1980s showed that an immunization regime that includes an initial live attenuated vaccine followed by an inactivated IB vaccine induces significant immune protection against diminished egg production [24,25,26]. High levels of hemagglutination-inhibiting antibodies against Massachusetts (Mass) IBV serotype provided better protection against drops in egg production post-challenge [26]. Regarding homologous immunization, the vaccination of growing pullets with two IBV live attenuated vaccines containing a Mass serotype followed by boosting with an inactivated vaccine containing a homologous Mass serotype of IBV resulted in significant protection against a Mass challenge at 61-week-old; however, it did not protect against an Arkansas (Ark) challenge at 52-weekold [27]. Furthermore, inclusion of the inactivated IB vaccines in the immunization schedule of layers induced higher serum neutralizing antibodies against some IBV challenge strains [28].

In young chicks, two antigenically different live-attenuated IBV vaccines have been used successfully in a vaccination program to combat diseases associated with recent IBV variants [29,30]. As a consequence, a variety of live-attenuated vaccines have been used during rearing (pullet period), and an oil-adjuvanted inactivated vaccine containing the Mass antigen has been injected prior to lay to broaden protection. There is evidence of a high level of antibody production, indicating that long-term protection is likely to occur against some IBV variants in long-lived birds (layers) [29]. Furthermore, the application of heterologous live-attenuated IBV vaccines in pullets followed by an oil-adjuvanted inactivated IBV vaccine prior to lay has already been proven to be effective against challenge with various strains of IBV in laying hens [28,31]. The long-lasting immune response of this vaccination regime in layers can be explained by the fact that a prolonged immune response can be achieved by following primary vaccination up with secondary vaccination every 2 to 4 weeks with live vaccines and then boosting with additional inactivated vaccines [32].

IBV serotypes are distributed differently across different regions in Canada. The majority of current IBV outbreaks in Eastern Canada are caused by the Delmarva (DMV/1639) and 4/91 serotypes [33,34]. Meanwhile, the Mass and Connecticut (Conn) variants are the predominant strains in Western Canada [35]. Beginning in 2010, Western Canada egg layer flocks experienced a sudden drop in egg production and an increase in shell-less eggs in a phenomenon referred to shell-less egg syndrome (SES), which is caused by the Mass serotype of IBV [35]. Therefore, it was necessary to mitigate this issue through the application of vaccination regimes depending on the commercially available vaccines in Canada. Live attenuated vaccines (Mass and Conn) and inactivated vaccines (Mass and Ark) are the only types of IB vaccines licensed in Canada. The vaccination of Canadian table-egg layers begins at one day and continues every 4–6 weeks until they reach 16-week-old. Previously, we observed that a combination of live attenuated and inactivated IB vaccines in layers’ immunization programs can result in better systemic and local immune responses than live attenuated vaccines alone [36]. Therefore, we conducted a further IBV vaccination/challenge study to compare the efficacy of these two immunization strategies against Mass serotype IBV inducing SES; one strategy included live attenuated vaccines containing Mass and Conn serotypes plus inactivated vaccine with a Mass serotype, and the other contained only live attenuated vaccines.

## 2. Materials and Methods

### 2.1. Ethical Statement

All the experimental procedures in this study were ethically approved by the University of Calgary’s Health Sciences Animal Care Committee (HSACC) and the Veterinary Sciences Animal Care Committee (VSACC) with the protocol number AC19-0113.

### 2.2. Layer Chickens

A total of one hundred and twenty 1-day-old specific-pathogen-free (SPF) chicks (White Leghorn) were purchased from the Canadian Food Inspection Agency (CFIA) (Ottawa, Ontario, Canada). The day-old SPF chicks were sexed based on a molecular assay described previously [37]. The SPF female chicks (*n* = 50) were reared in the University of Calgary’s Veterinary Science Research Station (VSRS). The birds were housed on deep litter with strict biosecurity measures, and they received water and feed ad libitum for the duration of the experiment. During the laying period, a light schedule of sixteen hours of light:eight hours of darkness was maintained.

### 2.3. IBV Challenge Strain and Vaccines

In this study, we challenged the birds with a Mass IBV isolate designated as 15AB-01 in its fourth serial passage through SPF embryonated chicken eggs (ECE). The Mass IBV isolate was isolated from tissue samples from a Western Canadian layer flock with a history of SES [35]. The 50% embryo infectious dose (EID_50_) of the virus was determined from 9-day-old SPF ECE following Reed and Muench’s method [38]; the virus titer was 10^8^ EID_50_/mL. Two commercial live attenuated IB vaccines were employed; one was composed of the Mass serotype (Merial Inc., Athens, GA, USA) and the other contained the Mass and Conn serotypes (Zoetis Inc, Kalamazoo, MI, USA). Each vial of the live vaccine was reconstituted in 30 mL of sterile phosphate-buffered saline (PBS; Life Technologies Corporation, Grand Island, NY, USA) to be administrated via eye drop according to the manufacturer’s instructions (30 µL per bird). The commercial inactivated vaccine used for this trial was BRON-NEWCAVAC^®^ SE, which contained the Mass serotype of IBV (Merck Animal Health, Division of Intervet, Inc., Omaha, NE, USA), and it was given intramuscularly (I/M) based on the manufacturer’s instructions (0.5 mL per bird).

### 2.4. Experimental Design

The design is presented in Table 1. Following sexing, fifty female chicks were initially divided into vaccinated (*n* = 36) and mock-vaccinated (*n* = 14) groups in two separate negative pressure rooms. At 14-week-old, the vaccinated group was randomly and evenly subdivided into two groups (L+LK and L+L), and each group was kept in a separate room. In terms of immunization, all birds in the vaccinated groups were primed with a Mass serotype live attenuated vaccine via eye drop at 1-day-old and then boosted with live attenuated vaccine containing the Mass and Conn serotypes at 2-, 5-, and 9-week-old. At 14-week-old, the L+LK group was vaccinated with a combination of live attenuated IB vaccine containing the Mass and Conn serotypes and a Mass serotype inactivated vaccine, while the L+L group was only vaccinated with live attenuated IB vaccine. Simultaneously, the mock-vaccinated (MV) group was administrated a placebo (PBS) via eye drop. At 26-week-old (peak of lay), the MV, L+LK, and L+L groups were further subdivided into MV/MI and MV/I; L+LK/MI and L+LK/I; and L+L/MI and L+L/I denoting mock-infected and infected groups, respectively. The hens in the L+LK/I (*n* = 10), L+L/I (*n* = 10), and MV/I (*n* = 9) groups were infected with a total volume of 150 µL containing 1 × 10^6^ EID_50_ Mass IBV isolate (15AB-01) per hen through the oculo-nasal route. Meanwhile, the birds in the MV/MI (*n* = 5), L+LK/MI (*n* = 8), and L+L/MI (*n* = 8) group were mock-infected oculo-nasally with 150 µL of PBS.

After infection, all birds were observed for clinical signs, egg production, and egg quality for 14 days post-infection (dpi). Oropharyngeal (OP) and cloacal (CL) swabs were also collected at 3, 7, and 14 dpi to detect viral shedding. Then, the swabs were preserved in PBS supplemented with 2% fetal calf serum and 2% penicillin and streptomycin (Gibco, Carlsbad, CA, USA), aliquoted, and stored at −80 °C until processing. At 5 dpi, 2 mL of blood was collected from the wing vein of all hens and kept in tubes containing anticoagulant to isolate the peripheral blood mononuclear cells (PBMCs). At 3 weeks post last vaccination, as well as at 10 and 14 dpi, 1 mL of blood was collected in plain tubes from all birds to monitor serum anti-IBV antibodies. Following 1 h at room temperature, the blood samples were centrifuged at 2000× *g* for 10 min (min) at 4 °C, and the serum was separated, aliquoted, and kept at −20 °C. At 14 dpi, all hens were euthanized via over-inhalation of isoflurane anesthesia followed by cervical dislocation, and then a post-mortem examination was performed to observe any gross lesions. Tissue specimens from the trachea, lung, kidney, cecal tonsil (CT), ovary, and different parts of the oviduct (magnum, isthmus, and uterus) were collected and preserved in RNA Save^®^ (Biological Industries, Beit Haemek, Israel) for IBV genome load quantification. Other parts from the ovary and different regions of oviduct (oviduct, the magnum, isthmus, and uterus) were fixed in 10% neutral buffered formalin (VWR International, Edmonton, AB, Canada) for histopathological examination.

### 2.5. Scoring of Clinical Signs and Measuring of Egg Parameters

Following the IBV challenge, the clinical signs in all groups were observed twice a day, and they were scaled according to their severity into four scores (from 0 to 3) using a previously described method [39]. Briefly, a score of 1 was given to non-specific signs such as ruffled feathers, huddling together near a heat source, depression with lowered head, and droopy wings. The severity of respiratory signs was scored as follows: a score of 0 denoted the absence of respiratory signs; a score of 1 denoted mild signs, where respiration was increase but beaks remained closed; a score of 2 denoted moderate signs, including increased respiration with open beaks, coughing, sneezing, watery eyes, and nasal discharge; a score of 3 denoted severe signs, such as obvious gasping. The overall clinical sign scores observed per day were recorded and calculated according to the aforementioned scoring system.

The collected eggs were analyzed daily up to 2 weeks post-infection (p.i.). All eggs were examined for external egg quality parameters such as egg length (L) and egg width (W), which were measured using a digital caliper with a minimum value 0.01 mm. Based on these measurements, the egg shape index (SI) was calculated following a formula previously described [40]. For internal egg quality, the albumen height and egg weight were measured for individual eggs using a digital caliper and scale, respectively. We calculated the Haugh units (HU) for each egg using a previously published formula [41]. The external and internal egg quality abnormalities among the collected eggs in all groups were recorded daily during the experimental period. Egg production performance was analyzed as the percentage of egg production/group/1 week period.

### 2.6. Monitoring of Serum Anti-IBV Antibodies

A commercial enzyme-linked immunosorbent assay (ELISA) kit (IDEXX Laboratories, Inc., Westbrook, ME, USA) was used to quantify anti-IBV antibodies in serum samples. The procedure was performed and the data were analyzed following the manufacturer’s instructions. The positive IBV samples had titers > 396 (cut-off).

### 2.7. Flow Cytometry Technique

The PBMCs were isolated from the collected blood at 5 dpi using a previously de-scribed procedure [42]. The PBMCs were adjusted to 2 × 10^6^ cells per well in 96-well U-bottom microtiter plates (Nunc A/S, DK, Roskilde, Denmark). Then, the cells were washed in a buffer composed of 1% bovine serum albumin (BSA) (Sigma-Aldrich, Saint Louis, MO, USA) in PBS. The plate was centrifuged at 233× *g* for 5 min at 4 °C, and the supernatant was discarded. The fragment crystallizable (Fc) region of the antibody was quenched via the resuspension of cells in 0.2% chicken serum diluted in 1% BSA for 15 min at 4 °C. After spinning the cells as mentioned above, the cell pellets were stained with a suspension of two monoclonal antibodies: mouse anti-chicken CD8-APC (Southern Biotech, Birmingham, AL, USA) and mouse anti-chicken CD4-PE (Southern Biotech, Birmingham, AL, USA) for 20 min at 4 °C in the dark. The controls were their relevant isotypes (APC and PE labelled), and the unstained control was 1% BSA. Following two washings in 1% BSA, 1% paraformaldehyde solution (Electron Microscopy Sciences, Hatfield, PA, USA) was used to fix the stained PBMCs. Finally, the samples were submitted to the University of Calgary’s Flow Cytometry Core Facility (Calgary, AB, Canada) for analysis. The data were generated using BD FACSDiva^TM^ 6.1.3 software (BD Bioscience, San Jose, CA, USA).

### 2.8. RNA Extraction and IBV Genome Load Quantification

The viral ribonucleic acid (RNA) was extracted from different tissues and swabs using Trizol^®^ reagent (Invitrogen Canada Inc., Burlington, ON, Canada) according to the manufacturer’s instructions. In order to measure the concentration of extracted RNA, a Nanodrop 1000 spectrophotometer (ThermoScientific, Wilmington, DE, USA) was used. A total of 1000 ng and 2000 ng of the obtained RNA were used to synthesize the cDNA following the manufacturer’s guidelines using random primers (High-Capacity Reverse Transcription Kit™, Applied Biosystems, Invitrogen Canada Inc., Burlington, ON, Canada) for the swabs and tissues, respectively. The quantification of the viral RNA was performed via a SYBR green real time quantitative polymerase chain reaction (RT-qPCR) assay using IBV nucleocapsid (N) gene-specific primers as previously described [43]. A standard curve was generated using five ten-fold serial dilutions (from 10^7^ to 10^3^) of N-gene plasmid prepared in-house to quantify the IBV genome copies [43]. The limit of detection of the SYBR green real time RT-qPCR was assessed using cycle-threshold (Ct) values for each reaction containing from 10^7^ to 10^3^ copies of the standard plasmids. A plot of threshold cycle (Ct) values against standard curve data was made to determine the limit of detection of this RT-qPCR assay. The detection limit of RT-qPCR refers to the lowest concentration of viral particles that can be detected.

### 2.9. Histopathology

The formalin-fixed tissue specimens from the ovary and different parts of oviduct (magnum, isthmus, and uterus) were submitted to the Diagnostic Services Unit (DSU) at the University of Calgary, Faculty of Veterinary Medicine, for further processing and to obtain hematoxylin and eosin (H & E)-stained sections. We examined the slides under a light microscope (Nikon ECLIPSE 50i, Tokyo, Japan) and found presumptive IBV-induced lesions (Table 2), which were scored according to a modified scoring system previously designed by Benyeda et al. [44]. Briefly, the lesions were scored as follows: (0) no change, (1) mild, (2) moderate, and (3) severe, as reported in Table 2.

### 2.10. Data Analysis

The Pearson’s chi-squared test was performed to compare the percentage of weekly egg production performance between the groups. Whereas, the egg quality parameters (SI and HU), clinical signs scores, serum anti-IBV antibodies at post-challenge, and IBV genome loads in OP and CL swabs were analyzed at different time points using the mixed-effects model. In order to identify the group differences, Tukey’s multiple comparisons test was employed. The differences among the groups in other parameters (CD4+ and CD8+ cell percentages, IBV genome loads in tissues, serum anti-IBV titers at pre-challenge, and the histopathological lesion scores) were identified using the Kruskal–Wallis test followed by Dunn’s multiple comparison test. The normality was assessed by D’Agostino-Pearson normality test before the analysis of data. All statistical tests and graphs were conducted and created, respectively, in GraphPad Prism 9.2.0 Software (GraphPad Prism Software, San Diego, CA, USA).

## 3. Results

### 3.1. Clinical Signs

**Figure 1:**
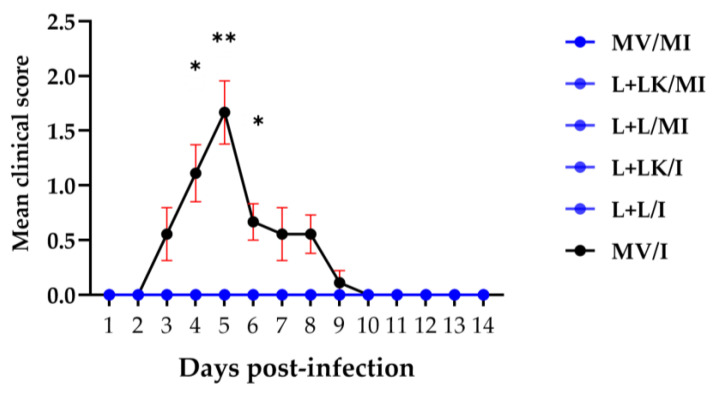
The daily mean clinical scores were monitored and calculated during the experimental period following infection with Mass IBV isolate (15AB-01). The mixed-effects model followed by Tukey’s multiple comparisons test was used to identify the group differences, and error bars represent the standard error of the mean (SEM). Asterisks indicate significant differences (* *p* < 0.05, ** *p* < 0.01). The blue-colored symbols refer to all groups except the mock-vaccinated infected (MV/I) group, which didn’t exhibit clinical signs.

Prior to the necropsy examination at 14 dpi, all hens were scored for respiratory and non-specific signs. Overall, the birds in the MV/I group exhibited clinical signs starting at 3 dpi and subsiding at 10 dpi (Figure 1). On the other hand, the mock-infected groups (MV/MI, L+LK/MI, and L+L/MI) and the vaccinated challenged groups (L+LK/I and L+L/I) showed no clinical signs throughout the experiment. In terms of daily mean clinical signs scores, both vaccinated challenged groups (L+LK/I and L+L/I) showed significantly lower scores than those calculated in the MV/I group at 4 dpi (*p* < 0.05), 5 dpi (*p* < 0.01), and 6 dpi (*p* < 0.05), thus indicating a significant protection against the clinical signs induced by Mass IBV challenge among these groups. Primary respiratory distress signs were observed (coughing, sneezing, and open beak breathing), as well as non-specific signs including depression and ruffled feathers were detected.

### 3.2. Egg Production and Egg Quality Parameters

**Figure 2:**
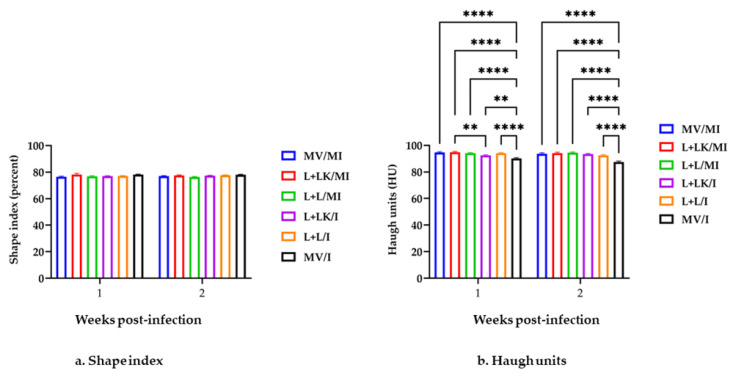
Shape index percent (**a**) and Haugh units (**b**) were measured for two weeks following infection with Mass IBV isolate (15AB-01). Mixed-effects model followed by Tukey’s multiple comparisons test was employed to detect the group differences. Error bars represent SEM. Asterisks indicate significant differences (** *p* < 0.01, **** *p* < 0.0001).

Over the first- and second-week post-challenge, egg production was not significantly different (*p* > 0.05) among all the treatment groups (data not shown). Regarding external egg quality, the SI exhibited no significant differences between all groups over two weeks post-infection (*p* > 0.05; Figure 2a). The SI of eggs in the MV/I group was more rounded (SI~78%) than the other groups over the entire experiment. There were no shell abnormalities in the mock-infected groups or L+LK/I group post-challenge. Most eggshell disorders were detected in the MV/I group from 3 dpi onwards and to a lesser degree in the L+L/I group. In total, the MV/I group yielded one egg with a soft shell, two eggs with cracked shells, two eggs with thin shells, three eggs with rough shells, and two eggs with hair cracks on the shell. The L+L/I group showed only two eggs with rough shells and three eggs with thin shells. In terms of internal egg quality, the HU in the MV/I group were significantly lower (*p* < 0.0001) than those of the mock-infected groups at the first and second weeks following IBV infection (Figure 2b). Furthermore, the HU in the L+L/I and L+LK/I groups were significantly higher (*p* < 0.0001 and *p* < 0.01, respectively) than those of the MV/I group at the 1st week of IBV infection; however, the L+LK/I group showed significantly lower (*p* < 0.01) HU compared to the L+LK/MI group at the same time point. At the second week of IBV infection, the HU in the L+LK/I and L+L/I groups were significantly higher (*p* < 0.0001) than the MV/I group. Over the two weeks p.i., 2 eggs with watery albumen were detected in the MV/I group at 6 and 8 dpi, whereas in the L+L/I group, only one egg with watery albumen was detected at 12 dpi.

### 3.3. Antibody-Mediated Immune Response

**Figure 3:**
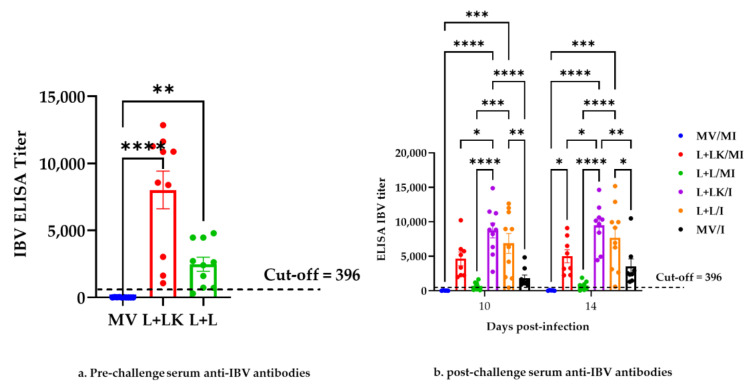
Anti-IBV antibody titers in serum collected at pre-challenge (3 weeks post last vaccination) (**a**) and post-challenge (**b**) at 10 and 14 days following infection with Mass IBV isolate (15AB-01). The mean titer differences between the groups at the pre-challenge period were statistically analyzed using the Kruskal–Wallis test followed by Dunn’s multiple comparison test. At post-challenge, the mean titers were compared using the mixed-effects model followed by Tukey’s multiple comparisons test. Error bars represent SEM. Asterisks indicate significant differences (* *p* < 0.05, ** *p* < 0.01, *** *p* < 0.001, **** *p* < 0.0001).

The mean measurable serum anti-IBV antibody titers at pre-challenge (3 weeks post last vaccination) and post-challenge (10 and 14 dpi) are shown in Figure 3a,b. As for the seroconversion of birds, both vaccinated groups (L+LK, L+L) seroconverted except for one bird in the L+L group during the pre-challenge period. All hens within all groups seroconverted after the IBV challenge, with the exception of three hens in the L+L/MI group. There was no seroconversion in any chickens in the MV and MV/MI groups throughout the study.

Statistically, both vaccinated groups (L+LK and L+L) had higher serum anti-IBV titers than the mock-vaccinated group (MV) (*p* < 0.0001 and *p* < 0.01, respectively) at the pre-challenge period.

In terms of antibody titers post-challenge, the titers in the L+LK/I group were significantly higher than in the MV/MI group (*p* < 0.0001), the L+LK/MI group (*p* < 0.05), and the L+L/MI group (*p* < 0.0001) at 10 and 14 dpi. Likewise, the anti-IBV antibody concentrations in the L+L/I group were significantly higher (*p* < 0.001) than in the MV/MI group and the L+L/MI group at 10 and 14 dpi. Compared to the L+LK/I and L+L/I groups at 10 and 14 dpi, the serum anti-IBV antibody titers in the MV/I group were significantly lower (*p* < 0.0001 and *p* < 0.01, *p* < 0.01 and *p* < 0.05, respectively). The anti-IBV antibody titers were not significantly different (*p* ˃ 0.05) between the L+LK/I and L+L/I groups throughout the study. There were no statistically significant differences (*p* ˃ 0.05) between the mock-infected groups; however, the anti-IBV titers in the L+LK/MI group were significantly higher (*p* < 0.05) than in the MV/MI group at 14 dpi.

### 3.4. Peripheral Blood T-Cell Subsets (CD4 + and CD8 + T Cells)

**Figure 4:**
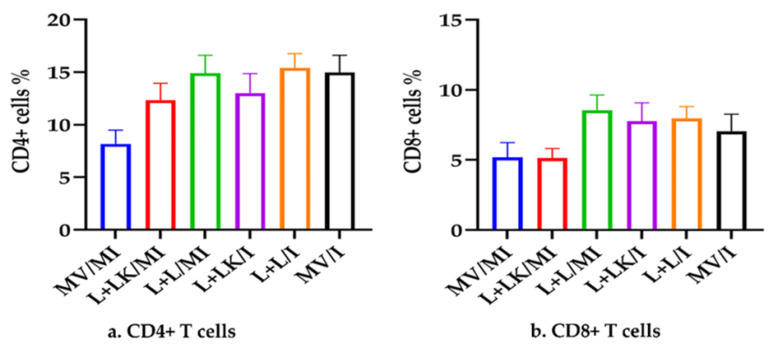
Percentage of CD4+ (**a**) and CD8+ (**b**) T cells in PBMCs isolated from blood at 5 days following infection with Mass IBV isolate (15AB-01). The Kruskal–Wallis test followed by Dunn’s multiple comparison test was used to compare the mean percentages of CD4+ and CD8+ T cells among the groups. Error bars represent SEM. Blue-colored columns refer to the mock-vaccinated mock-infected (MV/MI) group; red-colored columns indicate the live-vaccinated/inactivated-vaccinated mock-infected (L+LK/MI) group; green-colored columns show the live-vaccinated mock-infected (L+L/MI) group; purple-colored columns refer to the live-vaccinated/inactivated-vaccinated infected (L+LK/I) group; orange-colored columns show the live-vaccinated infected (L+L/I) group; black-colored columns refer to the mock-vaccinated infected (MV/I) group.

The gating strategy used to identify the T cell subsets (CD4+ and CD8+) among the other cell populations in PBMCs is shown in Figure S1. Briefly, the debris-free gating was initially performed using forward angle scatter (FSC) and side angle scatter (SSC). Then, the doublets were removed from the cells-free debris using FSC-H (height) versus FSC-A (area) gating, followed by SSC-H versus SSC-A gating. Singlets were categorized into CD4+ T cells, CD8+ T cells, CD4/CD8 double negative T cells (CD4/CD8 DN T cells), CD4/CD8 double positive T cells (CD4/CD8 DP T cells). The level of non-specific binding of PE-labelled anti-chicken CD4 and APC-labelled anti-chicken CD8 was 0% (Figure S2). They were assessed using isotype controls conjugated with PE and APC, respectively. The specific quantifications of CD4+ and CD8+ T cells using mouse anti-chicken CD4-PE and mouse anti-chicken CD8-APC are presented in Figure S2. The percentages of CD4+ and CD8+ T cells outputted by the flow cytometry analysis among the treated groups are depicted in Figure 4a,b, respectively. There was no significant difference in the CD4+ T-cell percentages at 5 dpi between the groups (*p* > 0.05). Similarly, the percentages of CD8+ T cells showed no statistical difference between the groups at 5 dpi (*p* > 0.05).

### 3.5. IBV Genome Loads

#### 3.5.1. IBV Genome Loads in Oropharyngeal and Cloacal Swabs

**Figure 5:**
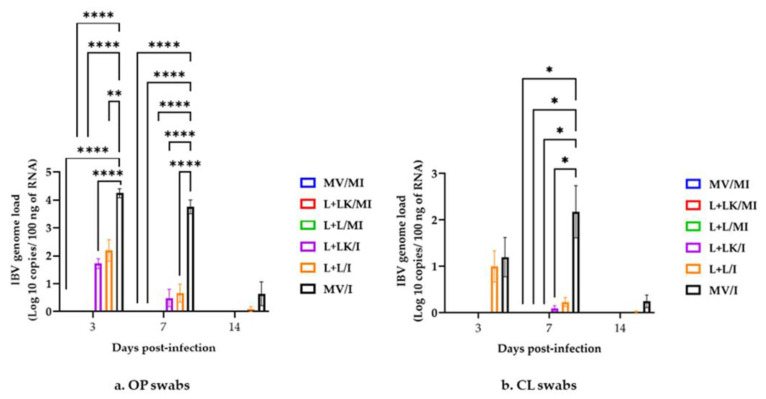
IBV genome loads were quantified from the OP (**a**) and CL (**b**) swabs collected at 3, 7, and 14 days following infection with Mass IBV isolate (15AB-01). The mixed-effects model followed by Tukey’s multiple comparisons test was used to detect the group differences. Error bars represent SEM. Asterisks indicate significant differences (* *p* < 0.05, ** *p* < 0.01, **** *p* < 0.0001). The detection limit of RT-qPCR was 0.8 log_10_ copies/100 ng of RNA.

The IBV genome loads in OP and CL swabs are shown in Figure 5a,b, respectively. The mock-infected groups exhibited no detectable levels of IBV genome loads in the swabs throughout the study. In terms of the OP swabs, the IBV genome loads of the MV/I group were significantly higher (*p* < 0.0001) than those observed in the mock-infected groups at 3 and 7 dpi. The MV/I also had a significantly higher IBV genome load than that quantified in the L+LK/I group at 3 and 7 dpi (*p* < 0.0001), and in the L+L/I group at 3 and 7 dpi (*p* < 0.01 and *p* < 0.0001, respectively). At 3 dpi, the IBV genome loads in the L+L/I and L+LK/I groups were significantly higher when compared to the mock-infected groups (*p* < 0.01) (pairwise comparison not shown). The IBV genome loads in the L+LK/I and L+L/I groups showed no significant difference (*p* > 0.05) throughout the observation period. At 14 dpi, there was no significant difference (*p* > 0.05) between all treated groups. In terms of the CL swabs, the IBV genome loads in the MV/I group were significantly higher (*p* < 0.05) than those observed in the mock-infected groups and the L+LK/I group at 7 dpi. There were no significant differences (*p* > 0.05) detected between all groups at 3 and 14 dpi.

#### 3.5.2. IBV Genome Loads in Tissues

**Figure 6:**
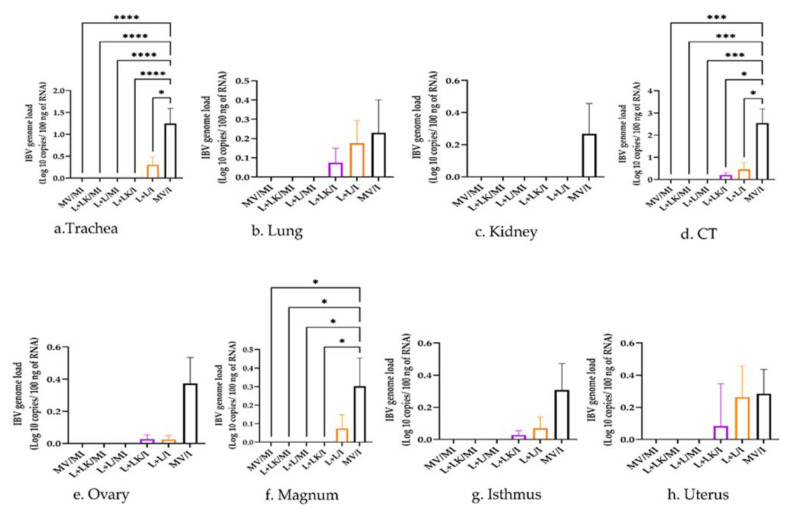
IBV genome loads were quantified in the trachea (**a**), lung (**b**), kidney (**c**), CT (**d**), ovary (**e**), magnum (**f**), isthmus (**g**), and uterus (**h**) at 14 days following infection with Mass IBV isolate (15AB-01). The Kruskal–Wallis test followed by Dunn’s multiple comparison test was performed to compare the genome loads of the groups. Error bars represent SEM. Asterisks indicate significant differences (* *p* < 0.05, *** *p* < 0.001, **** *p* < 0.0001). The detection limit of RT-qPCR was 0.8 log_10_ copies/100 ng of RNA. Purple-colored columns refer to the live-vaccinated/inactivated-vaccinated infected (L+LK/I) group; orange-colored columns show the live-vaccinated infected (L+L/I) group; black-colored columns refer to the mock-vaccinated infected (MV/I) group.

The IBV genome loads quantified by RT-qPCR in tissues are shown in Figure 6. The mock-infected groups were negative for the IBV genome loads in all examined tissues. In the trachea, the genome loads in the MV/I group were significantly higher than those observed in the mock-infected groups (*p* < 0.0001), the L+LK/I group (*p* < 0.0001), and the L+L/I group (*p* < 0.05). In the case of CT, the MV/I group had significantly higher IBV genome loads when compared to the mock-infected groups (*p* < 0. 001), the L+LK/I group (*p* < 0.05), and the L+L/I group (*p* < 0.05). In the magnum, the IBV genome loads in the MV/I group were significantly higher (*p* < 0.05) than those of the other groups except the L+L/I group. The IBV loads in other tissues such as lung, kidney, ovary, isthmus, and uterus did not exhibit significant differences (*p* > 0.05) between the groups. Furthermore, there were no statistically significant differences in IBV genome loads between the L+LK/I and L+L/I groups in any of the tested tissues (*p* > 0.05).

### 3.6. Histopathological Findings

**Figure 7:**
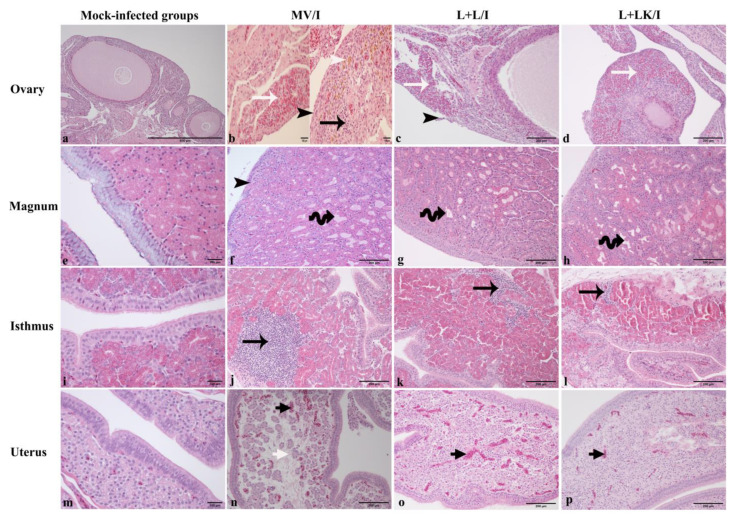
Histopathological lesions observed in ovary, magnum, isthmus, and uterus at 14 days following infection with Mass IBV isolate (15AB-01). (**a**,**e**,**i**,**m**) represent the mock-infected groups. (**b**,**f**,**j**,**n**) show the MV/I group. (**c**,**g**,**k**,**o**) refer to the L+L/I group. (**d**,**h**,**l**,**p**) indicate the L+LK/I group. Black arrowheads indicate sloughed epithelium; white arrowhead refers to hemosiderin laden macrophage; black arrows reveal mononuclear cell infiltrations; white arrows display the heterophils; coiled black arrows indicate dilated glands; short black and white arrows refer to congestion and edema, respectively.

The mock-infected groups demonstrated no obvious histopathological lesions in the ovary and different parts of the oviduct during the observation period (Figure 7a,e,i,m). Although no gross lesions were visible in all groups, there were microscopic changes in the IBV-infected groups.

In the MV/I group, the ovary showed focal sloughing of the ovarian epithelium, and the cortical stroma was infiltrated with either multi-focal heterophils or a few mononuclear cells. Furthermore, there were some aggregates of macrophages laden with hemosiderin and congested blood vessels in the cortical stroma (Figure 7b). In the magnum, the epithelia showed either attenuation or patchy areas of sloughing with deciliation, while the sub epithelial glands were dilated in some hens. The isthmus showed focal mononuclear cell infiltrations together with edema in the lamina propria. The uterus exhibited edema and congested blood capillaries underneath the epithelium (Figure 7f,j,n).

In the L+L/I, the ovary was characterized by sloughed epithelium, and the interstitial connective tissue of cortical stroma was filled with multiple areas of heterophilic cell infiltrations (Figure 7c), and the ovary in the L+LK/I revealed only random distributions of inflammatory cell infiltrations, mainly heterophils in the cortical stroma (Figure 7d). In the magnum, isthmus, and uterus of the L+L/I and L+LK/I groups, the epithelia and cilia were intact. However, there were some dilated glands in the lamina propria with edema, especially in the magnum, and the isthmus showed mononuclear cell infiltration underlying the epithelium in both groups. The uterus revealed only dilated and congested blood capillaries in the lamina propria in some birds of the L+LK/I and L+L/I groups (Figure 7g,h,k,l,o,p).

**Figure 8:**
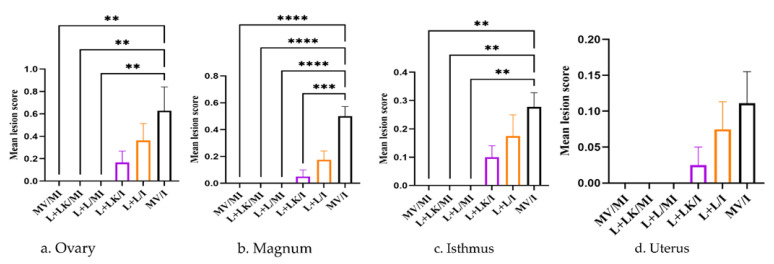
The mean lesion scores were calculated for ovary (**a**), magnum (**b**), isthmus (**c**), and uterus (**d**) at 14 days following infection with Mass IBV isolate (15AB-01). The Kruskal–Wallis test followed by Dunn’s multiple comparison test was used to identify the group differences. Error bars represent SEM. Asterisks indicate significant differences (** *p* < 0.01, *** *p* < 0.001, **** *p* < 0.0001). Purple-colored columns refer to the live-vaccinated/inactivated-vaccinated infected (L+LK/I) group; orange-colored columns show the live-vaccinated infected (L+L/I) group; black-colored columns refer to the mock-vaccinated infected (MV/I) group.

The histopathological lesion scores are shown in Figure 8. In the ovary and isthmus, the mean lesion scores in the MV/I group were significantly higher than those recorded in the mock-infected groups (*p* < 0.01). In the case of magnum, the MV/I group showed significantly higher lesion scores compared to those of the L+LK/I group (*p* < 0.001) and the mock-infected groups (*p* < 0.0001). However, the lesion scores were not significantly different between groups in the uterus (*p* > 0.05). Although the lesion scores varied between the L+L/I and L+LK/I groups in all tissues, no significant differences could be detected (*p* > 0.05).

## 4. Discussion

It was shown that the Mass IBV isolate (15AB-01) could experimentally reproduce egg production and egg quality deteriorations in SPF layer chickens in 2018, and this was in accordance with the field observations caused by the same isolate among Western Canada layer flocks in 2010 [35]. Therefore, we implemented two homologous vaccination strategies against Mass IBV isolate (15AB-01): the first contained inactivated IB vaccine and multiple boosters of live attenuated IB vaccines, and the second included only live IB vaccines. A ciliostasis or virus re-isolation test is the most commonly used method for evaluating IBV vaccine protection against challenge, particularly during vaccine licensing procedures [23]. Furthermore, clinical respiratory signs, histopathology, and virus detection by RT-qPCR have been also used as measures of vaccine protection following IBV challenge [45]. Similarly, the current vaccination regimes were evaluated using some parameters as clinical observations and egg production, histopathology of reproductive tissues, and virus detection in swabs and tissues by RT-qPCR.

Our results were three-fold. First, compared to the MV/I group, both vaccination strategies conferred a significant protection against clinical signs post-challenge, and they showed reduced viral shedding in OP and CL swabs. In addition, they revealed significantly lower genome loads in the trachea and CT. Furthermore, the vaccinated/challenged groups had higher serum anti-IBV antibody titers at 10 and 14 dpi and higher HU at the first and second week following IBV infection when compared to the MV/I group. Second, only the group that received live attenuated and inactivated IB vaccines exhibited a significant decrease in histopathological lesion scores and IBV genome loads in the magnum, as well as lower IBV genome loads in CL swabs at 7dpi when compared to those observed in the MV/I group. Third, there was no significant difference observed between the L+LK/I group and the L+L/I group on the basis of clinical signs, egg production and quality parameters, seroconversion, and genome load quantification following IBV challenge.

Previous studies have shown that using live attenuated IB vaccines combined with inactivated IB vaccines given just before the point of lay provide greater protection than the use of live vaccines alone [25,28,46]. Moreover, vaccination programs that include inactivated IB vaccines are more likely to induce a better anti-IBV antibody response in serum than those that only use live attenuated vaccines [28,36]; however, in our results, the serum anti-IBV titers were not significantly different between the L+LK/I and L+L/I groups at 10 and 14 dpi. Despite this, it is possible that significantly higher serum anti-IBV titers might have been observed in hens vaccinated with both live and inactivated vaccines as compared to those vaccinated with live vaccines alone if the study had continued for longer. This can be explained by the prolonged duration of the serum anti-IBV response by the use of an oil-adjuvanted inactivated vaccine associated with antigen persistence [47].

Types of IBV vaccine strains approved for use may vary between countries. The epidemiological knowledge of the regionally or locally predominant strains should guide this process. In Canada, the licensed live attenuated IB vaccines contain either Mass-type IBV alone (monovalent) or a combination of Mass and Conn strains of IBV (bivalent). While the inactivated vaccine involves either Mass-type IBV alone or a combination of Mass- and Ark-types of IBV. This enabled us to employ two homologous regimes containing Mass-type IBV against the relevant field IBV (Mass-type) that is commonly circulating among poultry flocks in the Eastern part of Canada [35]. Previous studies have shown that the best protection against field IBV challenge could be achieved by using a vaccine containing homologous strain [27,48]. In agreement, the current homologous vaccination regimes demonstrated a significant protection against the current IBV challenge. This was shown by complete absence of clinical respiratory illness, lower viral shedding from the swabs, and minimal histopathological lesion scores in the two vaccinated challenged groups compared to the mock-vaccinated challenged group.

In this study, the Mass IBV isolate (15AB-01) did not induce significantly lower egg production among challenged groups. This was not consistent with the previous experimental study that was conducted on the same IBV isolate [35]. Similarly, Chousalkar and Roberts [49] observed no significant decline in egg production following infection with Australian strains of IBV (T and N1/88) in laying hens; however, egg quality defects were detected. The possible explanations for this could be the difference in the route of infection, housing system, and number of birds used in this trial compared to the previous study [35]. The production of longer eggs may have been associated with a reduction in albumen quality after exposure to field strains of IBV [49]. In this context, the two vaccinated/challenged groups had significantly higher albumen heights with subsequent higher HU than those detected in the MV/I group. This was consistent with the significantly lowered IBV genome loads and histopathological lesion scores in the albumen forming region (magnum) in the L+LK/I group. It is likely that glandular dilation and the loss of mucopolysaccharides in the epithelium of the magnum contribute to the albumen defects [50]. Although the SI% showed no significant differences between the treatment groups, the eggs in the MV/I had a higher SI% than the other groups throughout the study. The differences in SI% were mainly impacted by the egg length rather than width. A previous study demonstrated changes in SI% after exposure to some IBV strains [49]; however, the alteration in SI% following IBV infection is not yet fully understood. Further investigation is required to clarify this.

There is controversy surrounding the precise role of antibodies in controlling IBV infections. A number of studies have shown that humoral immunity against IBV plays a key role in minimizing the clinical signs, histopathological lesions in some organs, and viral elimination [51,52]. Additionally, it has been reported that IBV-specific antibodies limit the spread of the virus from the trachea to other susceptible organs such as the kidneys and oviduct [9]. Our findings revealed significantly higher specific serum anti-IBV antibodies in the L+LK/I and L+L/I groups than that observed in the MV/I group. These significant antibody titers were accompanied by the absence of clinical signs, significantly lowered IBV genome loads in the swabs (OP at 3 and 7 dpi, CL at 7 dpi) and some tissues (trachea, CT, and magnum), and significant minimal lesion scores in the magnum. However, tissue protection is not closely correlated with serum antibody levels, but the local antibodies play a major role [53,54]. Likewise, we may speculate that the tissue protection, especially in the magnum of the L+LK/I group, may be due to the local antibodies detected in the reproductive tract tissue and wash.

The cell-mediated immune response represents the other arm of adaptive immune protection against IBV challenge, and its development is associated with reduced clinical severity, viral clearance, and rapid recovery from the disease [54,55]. CD8+ T cells are positively correlated with decreasing infection, and cytolysis of these cells mediates a reduction in clinical signs [56]. On the other hand, the CD4+ subset plays a critical role in virus infection through the activation and differentiation of virus-specific B cells [57]. However, we did not observe significant increases in CD4+ T cells in blood in response to vaccination or IBV infection similar to an antibody response. The CD8+ T cell responses occurred earlier than the serum IgG antibody responses to IBV in the blood and spleen [58], supporting the hypothesis that the cell-mediated immune response corresponded with decreased viral load and better clinical outcomes. It has also been shown that T cell activity induces the lysis of IBV (Gray strain)-infected cells in vitro. This lysis was mainly attributed to the effector T lymphocytes, mainly CD8+, while CD4+ cells had a lower impact [55]. Another study found that recipient chicks treated with donor CD8+ memory T cells were protected from acute IBV infection for the first 4 dpi and displayed mild clinical disease at 5 dpi [59]. Nevertheless, our observations showed that the CD8+ T cell percentages in PBMCs were not significantly higher in the L+LK/I and L+L/I groups at 5 dpi. Similarly, a recent IBV vaccination/challenge study was conducted on SPF laying hens and showed no significant difference in the peripheral CD8+ T cells between the vaccinated/challenged and non-vaccinated/challenged birds at 5 dpi [31]. This lack of group differences could be explained by infrequent blood sampling, as an increased frequency of blood sampling could have provided an opportunity to examine differences between experimental groups in terms of their T cell responses [60].

Our results showed that the Mass IBV isolate induced microscopic lesions in the reproductive tissues, which included epithelial and ciliary losses, inflammatory cell infiltrations, dilated glands, and some vascular changes such as congestion and edema. Most of the observed histopathological findings were noticeable in other studies using different IBV strains [8,61,62]. The presence of microscopic lesions confirmed the susceptibility of functional ovary and oviduct to the Mass IBV isolate (15AB-01) infection. In terms of reproductive lesion scores, the MV/I group showed significantly severe lesions than the mock-infected groups in all tissues except the uterus. Furthermore, the lesion scores in the MV/I did not display a significant difference when compared to L+LK/I and L+L/I groups in all tissues except magnum. The possible explanations for these scenarios could be the factors linked to IBV virulence such as the virus dose, route of infection, and age and breed of the birds.

Although the current study adds to the existing knowledge about the immunopathogenesis of the Mass IBV (15AB-01) infection in vaccinated and non-vaccinated layers, it has some limitations. One of these limitations is the lack of analysis of antigen specific T cell responses in PBMCs or tissues such as the reproductive tract. However, it can be argued that our results showed the presence of IBV-presumed microscopic lesions in the functional ovary and oviduct of challenged birds, so we speculated that some of the recruited T cells from circulation toward the site of injury may have been antigen-specific, as previously described [63]. Therefore, the detection of T cells in systemic circulation could be an acceptable parameter of cell-mediated immune responses against specific antigens. Furthermore, we relied on previously published experimental designs that have proposed to use the total T cell populations in the peripheral circulation as a parameter for T cell response against another IBV strain [31] or different viral infections affecting chickens [42,64]. Other limitations could be listed in this work such as the detection and quantification of IBV genome loads by RT-qPCR instead of the evaluation of infectious viral particles in tissues and swabs. It has been shown that the quantification of IBV genomes using RT-qPCR shows both replicating and non-replicating viruses [65]. Viral infectious particles could be assessed by virus isolation in embryonated chicken egg (ECE), tracheal organ culture (TOC), or cell culture [66]. However, the latter techniques are time-consuming, labor-intensive, and expensive. On the other hand, qPCR is quicker, easier, less costly, and very sensitive because it can detect viruses that are otherwise undetectable via virus isolation [67]. The high sensitivity of the qPCR assay enabled us to detect and quantify the potential low IBV genome loads in tissues such as the upper respiratory tract (trachea) at 2 weeks post-challenge. Various studies have shown that IBV genome loads in the trachea could peak at 3–5 days post-challenge and then decline rapidly to below the detected levels in the second week post-infection [12,68]. We can claim that the expected lower IBV titers in such tissue may be easily detected and quantified by RT-qPCR rather than using standard virus isolation assays at this time point.

## 5. Conclusions

In summary, the present study provides insights into the efficacy of two different vaccination strategies against the Mass IBV strain affecting Western Canada’s layer flocks. The current findings demonstrate that the two vaccination strategies conferred protection against Mass IBV despite the minor advantages of using the combination of live attenuated and inactivated IB vaccines that are licensed in Canada. Additionally, these results indicate that the vaccine strain could induce better protective efficacy against the challenge with homologous IBV strains. Further work is required to identify the development of reproductive tract lesions until recovery. This could be achieved by conducting a trial with a long experimental period. Furthermore, these vaccination regimes should be validated in the commercial layers.

## Figures and Tables

**Table 1:** The vaccination schedule and IBV challenge in the experimental groups.

Vaccination/Challenge				MV (*n* = 14)	L+LK (*n* = 18)	L+L (*n* = 18)
Live attenuated vaccine: 1-day-old (Mass) 2-, 5-, 9-, and 14-week-old (Mass + Conn)				No No	Yes Yes	Yes Yes
Inactivated IB vaccine (Mass) 14-week-old				No	Yes	No
At peak of lay	MV/MI (*n* = 5)	L+LK/MI (*n* = 8)	L+L/MI (*n* = 8)	MV/I (*n* = 9)	L+LK/I (*n* = 10)	L+L/I (*n* = 10)
Mass IBV challenge 26-week-old	No	No	No	Yes	Yes	Yes

**Table 2:** The lesions that were subjected to histopathological scoring.

Tissue	Microscopic Lesion
Ovary	Necrosis of ovarian covering epithelium Heterophilic and/or mononuclear cell infiltrations in the cortical stroma Congestion and edema in the cortical stroma
Magnum, isthmus, and uterus	Necrosis of lining epithelium and deciliation Mononuclear cell infiltrations in lamina propria Glandular dilatation Congestion and edema in the lamina propria

## Data Availability

Not applicable.

## Supplementary Materials

The following are available online at https://www.mdpi.com/article/10.3390/vaccines11020338/s1. Figure S1: Gating strategy that was performed to identify T cell subsets (CD4+ and CD8+) following staining of the peripheral blood mononuclear cells (PBMCs) by flow cytometry technique. Figure S2: Flow cytometry analysis for the quantification of CD4+ and CD8+ T cells from peripheral blood mononuclear cells (PBMCs) collected at 5 days following infection with Mass IBV isolate (15AB-01).

## References

[1] Cavanagh D. (2005). Coronaviruses in poultry and other birds. Avian Pathol..

[2] Cavanagh D. (2007). Coronavirus avian infectious bronchitis virus. Vet. Res..

[3] França M., Woolcock P.R., Yu M., Jackwood M.W., Shivaprasad H.L. (2011). Nephritis associated with infectious bronchitis virus Cal99 variant in game chickens. Avian Dis..

[4] Boroomand Z., Asasi K., Mohammadi A. (2012). Pathogenesis and tissue distribution of avian infectious bronchitis virus isolate IRFIBV32 (793/B serotype) in experimentally infected broiler chickens. Sci. World J..

[5] Liu S., Chen J., Han Z., Zhang Q., Shao Y., Kong X., Tong G. (2006). Infectious bronchitis virus: *S1* gene characteristics of vaccines used in China and efficacy of vaccination against heterologous strains from China. Avian Pathol..

[6] Ambali A.G., Jones R.C. (1990). Early pathogenesis in chicks of infection with an enterotropic strain of infectious bronchitis virus. Avian Dis..

[7] MacDonald J.W., McMartin D.A. (1976). Observations on the effects of the H52 and H120 vaccine strains of infectious bronchitis virus in the domestic fowl. Avian Pathol..

[8] Sevoian M., Levine P. (1957). Effects of Infectious Bronchitis on the Reproductive Tracts, Egg Production, and Egg Quality of Laying Chickens. Avian Dis..

[9] Raj G.D., Jones R.C. (1997). Infectious bronchitis virus: Immunopathogenesis of infection in the chicken. Avian Pathol..

[10] Bande F., Arshad S.S., Omar A.R., Hair-Bejo M., Mahmuda A., Nair V. (2017). Global distributions and strain diversity of avian infectious bronchitis virus: A review. Anim. Health Res. Rev..

[11] Moreno A., Franzo G., Massi P., Tosi G., Blanco A., Antilles N., Biarnes M., Majó N., Nofrarías M., Dolz R. (2017). A novel variant of the infectious bronchitis virus resulting from recombination events in Italy and Spain. Avian Pathol..

[12] de Wit J.J., de Jong M.C., Pijpers A., Verheijden J.H. (1998). Transmission of infectious bronchitis virus within vaccinated and unvaccinated groups of chickens. Avian Pathol..

[13] Cook J.K.A., Pattison P.F.M.M., Alexander J.M.B.D.J. (2008). Coronaviridae. Poultry Diseases.

[14] Jordan B. (2017). Vaccination against infectious bronchitis virus: A continuous challenge. Vet. Microbiol..

[15] Roh H.J., Hilt D.A., Williams S.M., Jackwooda M.W. (2013). Evaluation of infectious bronchitis virus Arkansas-type vaccine failure in commercial broilers. Avian Dis..

[16] De Wit J.J., Swart W.A., Fabri T.H. (2010). Efficacy of infectious bronchitis virus vaccinations in the field: Association between the alpha-IBV IgM response, protection and vaccine application parameters. Avian Pathol..

[17] Chhabra R., Forrester A., Lemiere S., Awad F., Chantrey J., Ganapathy K. (2015). Mucosal, Cellular, and Humoral Immune Responses Induced by Different Live Infectious Bronchitis Virus Vaccination Regimes and Protection Conferred against Infectious Bronchitis Virus Q1 Strain. Clin. Vaccine Immunol..

[18] Okino C.H., Alessi A.C., Montassier Mde F., Rosa A.J., Wang X., Montassier H.J. (2013). Humoral and cell-mediated immune responses to different doses of attenuated vaccine against avian infectious bronchitis virus. Viral Immunol..

[19] Bande F., Arshad S.S., Bejo M.H., Moeini H., Omar A.R. (2015). Progress and challenges toward the development of vaccines against avian infectious bronchitis. J. Immunol. Res..

[20] De Wit J.J., Boelm G.J., van Gerwe T.J., Swart W.A. (2013). The required sample size in vaccination-challenge experiments with infectious bronchitis virus, a meta-analysis. Avian Pathol..

[21] Awad F., Forrester A., Baylis M., Lemiere S., Ganapathy K., Hussien H.A., Capua I. (2015). Protection conferred by live infectious bronchitis vaccine viruses against variant Middle East IS/885/00-like and IS/1494/06-like isolates in commercial broiler chicks. Vet. Rec. Open.

[22] Bru T., Vila R., Cabana M., Geerligs H.J. (2017). Protection of chickens vaccinated with combinations of commercial live infectious bronchitis vaccines containing Massachusetts, Dutch and QX-like serotypes against challenge with virulent infectious bronchitis viruses 793B and IS/1494/06 Israel variant 2. Avian Pathol..

[23] De Wit J.J., Cook J.K. (2014). Factors influencing the outcome of infectious bronchitis vaccination and challenge experiments. Avian Pathol..

[24] Box P.G., Beresford A.V., Roberts B. (1980). Protection of laying hens against infectious bronchitis with inactivated emulsion vaccines. Vet. Rec..

[25] Box P.G., Ellis K.R. (1985). Infectious bronchitis in laying hens: Interference with response to emulsion vaccine by attenuated live vaccine. Avian Pathol..

[26] Box P.G., Holmes H.C., Finney P.M., Froymann R. (1988). Infectious bronchitis in laying hens: The relationship between haemagglutination inhibition antibody levels and resistance to experimental challenge. Avian Pathol..

[27] Muneer M.A., Newman J.A., Halvorson D.A., Sivanandan V., Coon C.N. (1987). Effects of avian infectious bronchitis virus (Arkansas strain) on vaccinated laying chickens. Avian Dis..

[28] De Wit J.J.S., Malo A., Cook J.K.A. (2019). Induction of IBV strain-specific neutralizing antibodies and broad spectrum protection in layer pullets primed with IBV Massachusetts (Mass) and 793B vaccines prior to injection of inactivated vaccine containing Mass antigen. Avian Pathol..

[29] De Wit J.J., Nieuwenhuisen-van Wilgen J., Hoogkamer A., van de Sande H., Zuidam G.J., Fabri T.H. (2011). Induction of cystic oviducts and protection against early challenge with infectious bronchitis virus serotype D388 (genotype QX) by maternally derived antibodies and by early vaccination. Avian Pathol..

[30] Terregino C., Toffan A., Beato M.S., De Nardi R., Vascellari M., Meini A., Ortali G., Mancin M., Capua I. (2008). Pathogenicity of a QX strain of infectious bronchitis virus in specific pathogen free and commercial broiler chickens, and evaluation of protection induced by a vaccination programme based on the Ma5 and 4/91 serotypes. Avian Pathol..

[31] Hassan M.S.H., Buharideen S.M., Ali A., Najimudeen S.M., Goldsmith D., Coffin C.S., Cork S.C., van der Meer F., Abdul-Careem M.F. (2022). Efficacy of Commercial Infectious Bronchitis Vaccines against Canadian Delmarva (DMV/1639) Infectious Bronchitis Virus Infection in Layers. Vaccines.

[32] Pulendran B., Ahmed R. (2011). Immunological mechanisms of vaccination. Nat. Immunol..

[33] Hassan M.S.H., Ojkic D., Coffin C.S., Cork S.C., van der Meer F., Abdul-Careem M.F. (2019). Delmarva (DMV/1639) Infectious Bronchitis Virus (IBV) Variants Isolated in Eastern Canada Show Evidence of Recombination. Viruses.

[34] Martin E.A., Brash M.L., Hoyland S.K., Coventry J.M., Sandrock C., Guerin M.T., Ojkic D. (2014). Genotyping of infectious bronchitis viruses identified in Canada between 2000 and 2013. Avian Pathol..

[35] Amarasinghe A., Popowich S., De Silva Senapathi U., Abdul-Cader M.S., Marshall F., van der Meer F., Cork S.C., Gomis S., Abdul-Careem M.F. (2018). Shell-Less Egg Syndrome (SES) Widespread in Western Canadian Layer Operations Is Linked to a Massachusetts (Mass) Type Infectious Bronchitis Virus (IBV) Isolate. Viruses.

[36] Buharideen S.M., Hassan M.S.H., Najimudeen S.M., Niu D., Czub M., Gomis S., Abdul-Careem M.F. (2021). Immune Responses in Laying Hens after an Infectious Bronchitis Vaccination of Pullets: A Comparison of Two Vaccination Strategies. Vaccines.

[37] He L., Martins P., Huguenin J., Van T.N., Manso T., Galindo T., Gregoire F., Catherinot L., Molina F., Espeut J. (2019). Simple, sensitive and robust chicken specific sexing assays, compliant with large scale analysis. PLoS ONE.

[38] Reed L.J., Muench H. (1938). A Simple Method of Estimating Fifty per Cent Endpoints12. Am. J. Epidemiol..

[39] De Silva Senapathi U., Abdul-Cader M.S., Amarasinghe A., van Marle G., Czub M., Gomis S., Abdul-Careem M.F. (2018). The In Ovo Delivery of CpG Oligonucleotides Protects against Infectious Bronchitis with the Recruitment of Immune Cells into the Respiratory Tract of Chickens. Viruses.

[40] Anderson K.E., Tharrington J.B., Curtis P.A. (2004). Shell Characteristics of Eggs from Historic Strains of Single Comb White Leghorn Chickens and the Relationships of Egg Shape to Shell Strength. Int. J. Poult. Sci..

[41] Monira K.N., Salahuddin M., Miah G. (2003). Effect of Breed and Holding Period on Egg Quality Characteristics of Chicken. Int. J. Poult. Sci..

[42] Barboza-Solis C., Najimudeen S.M., Perez-Contreras A., Ali A., Joseph T., King R., Ravi M., Peters D., Fonseca K., Gagnon C.A. (2021). Evaluation of Recombinant Herpesvirus of Turkey Laryngotracheitis (rHVT-LT) Vaccine against Genotype VI Canadian Wild-Type Infectious Laryngotracheitis Virus (ILTV) Infection. Vaccines.

[43] Kameka A.M., Haddadi S., Kim D.S., Cork S.C., Abdul-Careem M.F. (2014). Induction of innate immune response following infectious bronchitis corona virus infection in the respiratory tract of chickens. Virology.

[44] Benyeda Z., Szeredi L., Mató T., Süveges T., Balka G., Abonyi-Tóth Z., Rusvai M., Palya V. (2010). Comparative histopathology and immunohistochemistry of QX-like, Massachusetts and 793/B serotypes of infectious bronchitis virus infection in chickens. J. Comp. Pathol..

[45] Jackwood M.W., Jordan B.J., Roh H.J., Hilt D.A., Williams S.M. (2015). Evaluating Protection against Infectious Bronchitis Virus by Clinical Signs, Ciliostasis, Challenge Virus Detection, and Histopathology. Avian Dis..

[46] Sjaak de Wit J.J., Ter Veen C., Koopman H.C.R. (2020). Effect of IBV D1466 on egg production and egg quality and the effect of heterologous priming to increase the efficacy of an inactivated IBV vaccine. Avian Pathol..

[47] Coffman R.L., Sher A., Seder R.A. (2010). Vaccine adjuvants: Putting innate immunity to work. Immunity.

[48] Yan S., Zhao J., Xie D., Huang X., Cheng J., Guo Y., Liu C., Ma Z., Yang H., Zhang G. (2018). Attenuation, safety, and efficacy of a QX-like infectious bronchitis virus serotype vaccine. Vaccine.

[49] Chousalkar K.K., Roberts J.R. (2009). Effects of Australian strains of infectious bronchitis virus on internal and external quality of hen eggs. Anim. Prod. Sci..

[50] Butler E.J., Curtis M.J., Pearson A.W., McDougall J.S. (1972). Effect of infectious bronchitis on the structure and composition of egg albumen. J. Sci. Food Agric..

[51] Cook J.K., Davison T.F., Huggins M.B., McLaughlan P. (1991). Effect of in ovo bursectomy on the course of an infectious bronchitis virus infection in line C White Leghorn chickens. Arch. Virol..

[52] Cook J.K., Huggins M.B., Ellis M.M. (1991). Use of an infectious bronchitis virus and Escherichia coli model infection to assess the ability to vaccinate successfully against infectious bronchitis in the presence of maternally-derived immunity. Avian Pathol..

[53] Ignjatovic J., McWaters P.G. (1991). Monoclonal antibodies to three structural proteins of avian infectious bronchitis virus: Characterization of epitopes and antigenic differentiation of Australian strains. J. Gen. Virol..

[54] Raggi L.G., Lee G.G. (1965). Lack of Correlation between Infectivity, Serologic Response and Challenge Results in Immunization with an Avian Infectious Bronchitis Vaccine. J. Immunol..

[55] Collisson E.W., Pei J., Dzielawa J., Seo S.H. (2000). Cytotoxic T lymphocytes are critical in the control of infectious bronchitis virus in poultry. Dev. Comp. Immunol..

[56] Pei J., Sekellick M.J., Marcus P.I., Choi I.S., Collisson E.W. (2001). Chicken interferon type I inhibits infectious bronchitis virus replication and associated respiratory illness. J. Interferon Cytokine Res..

[57] Ahmed R., Butler L.D., Bhatti L. (1988). T4+ T helper cell function in vivo: Differential requirement for induction of antiviral cytotoxic T-cell and antibody responses. J. Virol..

[58] Liu G., Wang Q., Liu N., Xiao Y., Tong T., Liu S., Wu D. (2012). Infectious bronchitis virus nucleoprotein specific CTL response is generated prior to serum IgG. Vet. Immunol. Immunopathol..

[59] Pei J., Briles W.E., Collisson E.W. (2003). Memory T cells protect chicks from acute infectious bronchitis virus infection. Virology.

[60] Seo S.H., Collisson E.W. (1997). Specific cytotoxic T lymphocytes are involved in in vivo clearance of infectious bronchitis virus. J. Virol..

[61] Chousalkar K.K., Roberts J.R., Reece R. (2007). Comparative histopathology of two serotypes of infectious bronchitis virus (T and n1/88) in laying hens and cockerels. Poult. Sci..

[62] Pereira N.A., Alessi A.C., Montassier H.J., Pereira R.J.G., Taniwaki S.A., Botosso V.F., Rui B.R., Richtzenhain L.J. (2019). Gonadal pathogenicity of an infectious bronchitis virus strain from the Massachusetts genotype. Braz. J. Microbiol..

[63] Ghani S., Feuerer M., Doebis C., Lauer U., Loddenkemper C., Huehn J., Hamann A., Syrbe U. (2009). T cells as pioneers: Antigen-specific T cells condition inflamed sites for high-rate antigen-non-specific effector cell recruitment. Immunology.

[64] Rodenberg J., Sharma J.M., Belzer S.W., Nordgren R.M., Naqi S. (1994). Flow cytometric analysis of B cell and T cell subpopulations in specific-pathogen-free chickens infected with infectious bursal disease virus. Avian Dis..

[65] Klein D. (2002). Quantification using real-time PCR technology: Applications and limitations. Trends Mol. Med..

[66] De Wit J.J. (2000). Detection of infectious bronchitis virus. Avian Pathol..

[67] Roh H.J., Hilt D.A., Jackwood M.W. (2014). Detection of infectious bronchitis virus with the use of real-time quantitative reverse transcriptase-PCR and correlation with virus detection in embryonated eggs. Avian Dis..

[68] Raj G.D., Jones R.C. (1997). Effect of T-cell suppression by cyclosporin on primary and persistent infections of infectious bronchitis virus in chickens. Avian Pathol..

