# Supplementary material for: Efficacy of Two Vaccination Strategies against Infectious Bronchitis in Laying Hens
Source: Vaccines (Basel). 2023 Feb 2;11(2):338. doi: 10.3390/vaccines11020338 (PMC9967544; doi:10.3390/vaccines11020338)
Supplement: Supplementary file 1 [file vaccines-11-00338-s001.zip › vaccines-2126162-supplementary.pdf]

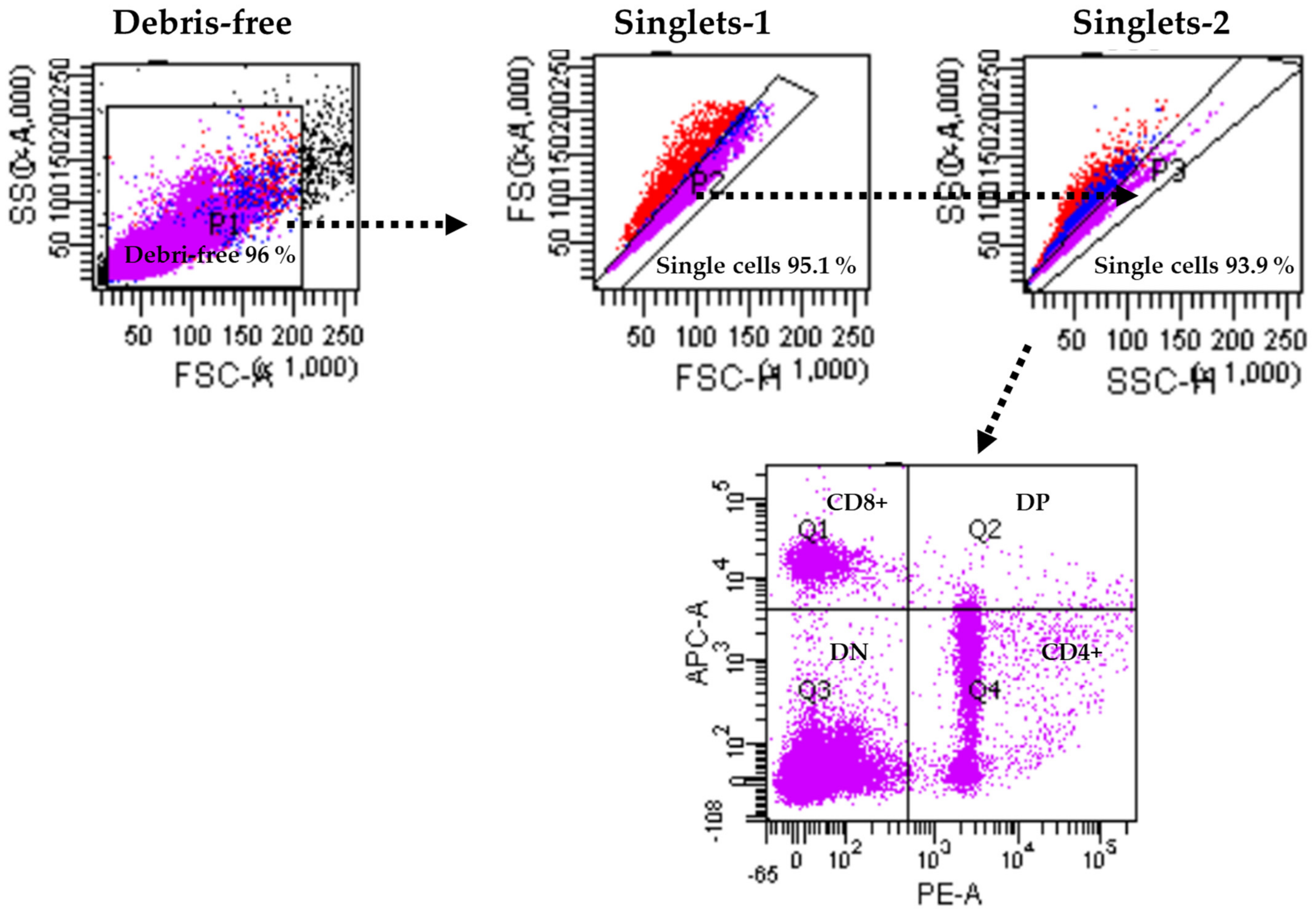

Figure S1. Gating strategy that was performed to identify T cell subsets (CD4+ and CD8+) following staining of the peripheral blood mononuclear cells (PBMCs) by flow cytometry technique. Firstly, the removal of debris from the stained cells was achieved by forward angle scatter (FSC) and side angle scatter (SSC). Next, the doublets were excluded from the cells-free debris using height and area parameters of FSC and SCC. Then, the obtained singlets were subdivided based on expression of CD4 and CD8 (expressed on T cells) into CD8+ T cells (Q1), CD4+ T cells (Q4), CD4/CD8 double positive T cells (Q2; DP), and CD4/CD8 double negative T cells (Q3; DN).

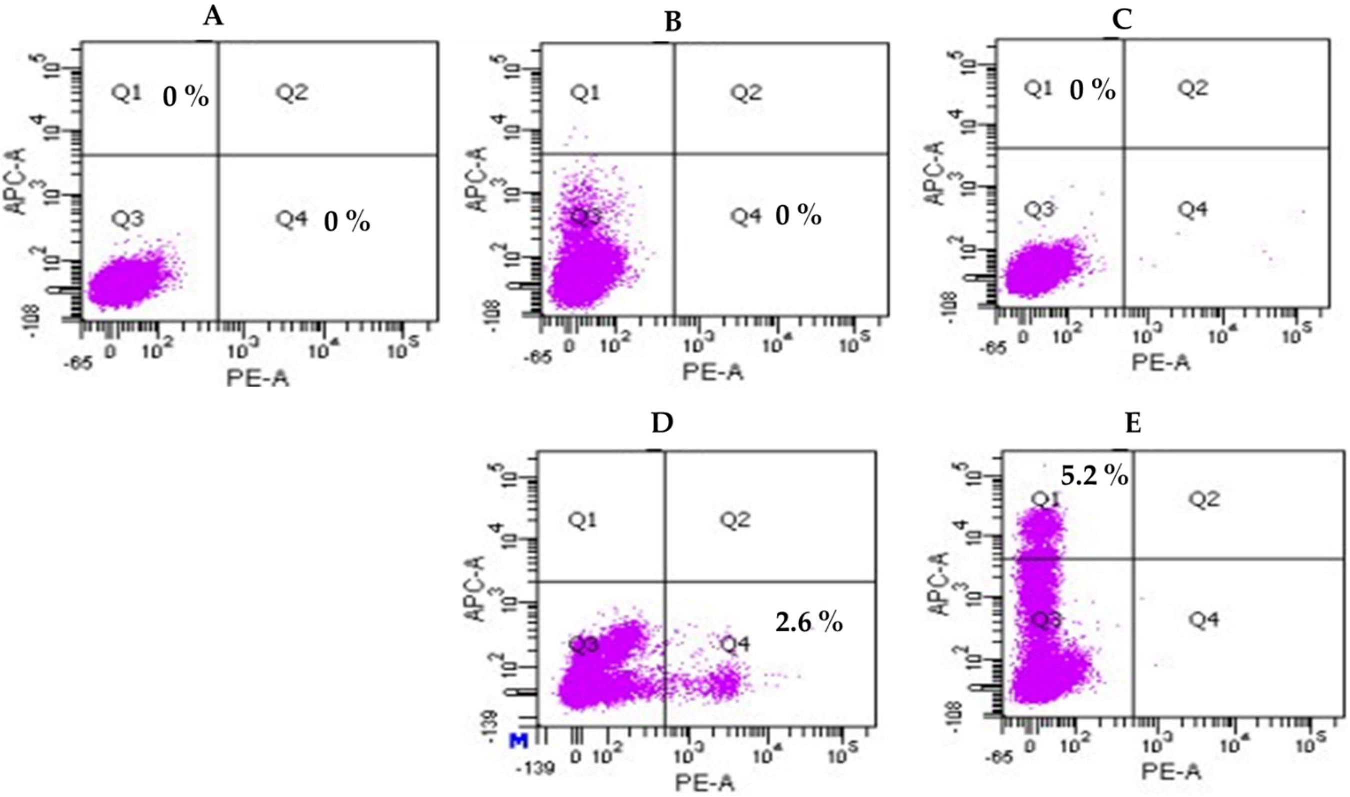

Figure ~~S1~~S2. Flow cytometry analysis for the quantification of CD4+ and CD8+ T cells from ~~peripheral blood mononuclear cells (~~PBMCs~~)~~ collected at 5 days following infection with Mass IBV isolate (15AB-01). A refers to fluorescence-activated cell sorting (FACS) plots of unstained control. B and C refer to FACS plots of isotype control for PE labelled mouse anti-chicken CD4 antibody and APC labelled mouse anti-chicken CD8 antibody, respectively. D and E show representative FACS plots of CD4+ and CD8+ T cells in PBMCs, respectively.
